# Hyperbranch-Crosslinked S-SEBS Block Copolymer Membranes for Desalination by Pervaporation

**DOI:** 10.3390/membranes10100277

**Published:** 2020-10-10

**Authors:** Mengyu Yan, Yunyun Lu, Na Li, Feixiang Zeng, Qinzhuo Wang, Hongcun Bai, Zongli Xie

**Affiliations:** 1 Shaanxi Key Laboratory of Energy Chemical Process Intensification, School of Chemical Engineering and Technology, Xi’an Jiaotong University, Xi’an 710049, China; ymy0356@stu.xjtu.edu.cn (M.Y.); Lu_Yunyun@163.com (Y.L.); feixiang_zeng@163.com (F.Z.); wdxo.621@163.com (Q.W.); 2 State Key Laboratory of High-Efficiency Utilization of Coal and Green Chemical Engineering, Ningxia University, Yinchuan 750021, China; hongcunbai@nxu.edu.cn; 3 CSIRO Manufacturing, Private Bag 10, Clayton South MDC, VIC 3169, Australia

**Keywords:** sulfonated styrene-ethylene/butylene-styrene copolymer, hyperbranched polyester, crosslinking, pervaporation, desalination

## Abstract

Sulfonated aromatic polymer (SAP) featuring hydrophilic nanochannels for water transport is a promising membrane material for desalination. SAPs with a high sulfonation degree favor water transport but suffer from reduced mechanical strength and membrane swelling. In this work, a hyperbranched polyester, H302, was introduced to crosslink a sulfonated styrene-ethylene/butylene-styrene (S-SEBS) copolymer membrane. The effects of crosslinking temperature and amount of H302 on the microstructure, and the pervaporation desalination performance of the membrane, were investigated. H302/S-SEBS copolymer membranes with different crosslinking conditions were characterized by various techniques including FTIR, DSC, EA, SEM, TEM and SAXS, and tensile strength, water sorption and contact angle measurements. The results indicate that the introduction of hyperbranched polyester enlarged the hydrophilic microdomain of the S-SEBS membrane. Crosslinking with hyperbranched polyester with heat treatment effectively enhanced the mechanical strength of the S-SEBS membrane, with the tensile strength being increased by 140–200% and the swelling ratio reduced by 45–70%, while reasonable water flux was maintained. When treating 5 wt% hypersaline water at 65 °C, the optimized crosslinked membrane containing 15 wt% H302 and heated at 100 °C reached a water flux of 9.3 kg·m^−2^·h^−1^ and a salt rejection of 99.9%. The results indicate that the hyperbranched-S-SEBS membrane is promising for use in PV desalination.

## 1. Introduction

Freshwater scarcity is considered the third global crisis after food and oil shortages, seriously threatening human lives and social development [1]. Desalination by membrane technology plays a key role in solving worldwide freshwater scarcity. Among various membrane desalination technologies, pervaporation (PV) desalination has received increasing attention due to its unique advantages in handling high-salinity water; notably, its energy demands are independent of salt concentrations [2,3,4]. In addition, high salt rejection can be achieved due to the high selectivity of the membrane, in addition to the nonvolatile nature of salt. The membranes employed in the PV desalination process cover a wide range of materials including polymers, zeolites, amorphous silica and two-dimensional nanomaterials [2]. Due to their good film-forming ability and potential for scale-up and industrialization, polymer materials have been most widely studied in membrane synthesis. However, the relatively low flux of polymer PV membranes remains an obstacle for industrialization. The development of high-performance membrane materials has therefore become a major challenge in the application of PV desalination. To date, much effort has been made to enhance the water flux of polymer membranes. Based on the solution-diffusion mechanism of PV desalination, membrane materials used in PV can be modified in terms of their hydrophilicity and diffusivity. Membrane hydrophilicity can be increased by enhancing hydrogen bonding or ion-dipole interactions between water molecules and the materials. Meanwhile, water diffusivity can be controlled by regulating the free volume of the polymers, the morphology of the nanochannels or the size of the hydrophilic domain [5,6].

Sulfonated aromatic polymer (SAP), whose active functional groups can form hydrophilic nanochannels for water transport, is a promising material for water desalination [7,8]. Sulfonated poly (styrene-ethylene/butylene-styrene) (S-SEBS) is a kind of SAP with an extremely hydrophobic backbone and abundant hydrophilic groups [9,10]. The micro phase separation from the two domains then leads to the formation of continuous nanochannels that are favorable for water diffusion [11]. Our previous work [12] reported the potential application of S-SEBS in PV desalination and indicated that a higher sulfonation degree tended to increase the water flux. However, excessive swelling and weaker mechanical properties were observed in highly sulfonated membranes. Crosslinking is often used to improve the mechanical properties of membranes. Introducing extra crosslinkers that can form a stable crosslinking network with the sulfonated groups is widely used to crosslink SAP [13]. Small molecules and linear polymers with functional groups have been widely studied as crosslinkers. For instance, Serge et al. [14] used polyols, e.g., ethylene glycol, glycerol and poly (vinyl alcohol) as crosslinkers; Nolte et al. [15] and Kaur et al. [16] used bis(4-aminophenyl) sulfone and carbonyldiimidazole as the coupling agent. Though the aforementioned attempts successfully enhanced the mechanical strength of the membrane, the hydrophilicity of the membrane was reduced significantly, which led to the reduction of the water flux. In addition, the small molecules were prone to be lost during long-term operation. Therefore, increasing mechanical stability without lowering the flux is the ultimate purpose of crosslinking. It has already been reported that by controlling the crosslinking degree and chain length of the crosslinking agent, essential membrane properties influencing the flux, such as water uptake, free volume and stability, can be manipulated [17]. 

The hyperbranched polymer (HBP) is an efficient crosslinking agent with a large number of active terminal groups for crosslinking; meanwhile, its special topology facilitates the transport of solvent molecules, which has potential in the trade-off phenomenon between flux and selectivity [18]. Hyperbranched polymers have demonstrated unique advantages in the modification of dense membranes such as nanofiltration membranes, reverse osmosis membranes and PV membranes.

Currently, the application of hyperbranched polymers in membranes has mostly concentrated on the tuning of microstructures and the optimization of chemical modifications. The branching structure of the hyperbranched polymer can break the dense stacking of polymer chains, increasing the free volume of the material; thus, the diffusion rate and permeation flux can be enhanced. Wei et al. [19] prepared hydroxyl-terminated hyperbranched polyester PV membranes crosslinked with glutaraldehyde (GA). The results showed that the loose molecular structure of the hyperbranched polyester significantly enlarged the free volume, and the crosslinked network structure enhanced the mechanical properties of the membrane simultaneously. Additionally, the remaining unreacted hydrophilic end groups offered the membrane better hydrophilicity. Luo et al. [20] crosslinked an ethylcellulose (EC) PV membrane with acrylic acid-modified hyperbranched polyester. When compared with membranes crosslinked with small molecules like ethylene glycol dmethylacrglate (EGDM), those crosslinked with hyperbranched polyester exhibited higher flux. Sun et al. [21] modified polyvinyl alcohol (PVA) with hyperbranched polyester (HBPE) for the separation of ethylene glycol and water using PV. The crosslinking structure resulted in a significant decrease of swelling in the membrane in water. The hydrogen bonding and steric hindrance between PVA and HBPE in the crosslinked network hindered the stacking of PVA polymer chains, accelerating the diffusivity of water. 

In this paper, we report on the modification of S-SEBS membranes using hydroxyl-terminated hyperbranched polyester H302 to enhance the mechanical strength of the membrane while maintaining the water flux for PV desalination. Hyperbranch-crosslinked S-SEBS copolymer membranes with different crosslinking degrees were prepared under different crosslinking conditions, i.e., crosslinking temperature and the amount of crosslinking agent used. The chemical composition of membranes was characterized by Fourier transform infrared spectroscopy (FTIR) and elemental analysis (EA). Tensile strength and thermogravimetry (TG) were used to investigate the mechanical and thermal stability. Water sorption and contact angle measurements were applied to investigate the hydrophilic properties of the crosslinked S-SEBS membranes. The microstructure and morphology were detected by small angle X-ray scattering (SAXS) and transmission electron microscopy (TEM), respectively. PV performance was evaluated concerning water flux and salt rejection of membranes with 5 wt% aqueous NaCl solution as the feed solution.

## 2. Materials and Methods

### 2.1. Materials

Sulfonated styrene-ethylene/butylene-styrene (S-SEBS) with a sulfonation degree of 60% was prepared according to the method described in our previous work [12]. Raw SEBS triblock copolymer (Kraton G1650 grade, molecular weight 50,000 Da) was purchased from Shell LLC, Detroit, MI, USA. The styrene block content of the polymer was 30 wt%. Hydroxyl-terminated hyperbranched polyesters H302 (molecular weight 2500 Da, hydroxyl numbers 10–12 mol·mol^−1^) were supplied by Wuhan Hyperbranched Polymer Resins Science & Technology Co., Ltd, Wuhan, China. Other chemicals, including tetrahydrofuran, n-hexanol, sodium hydroxide, phenolphthalein and sodium chloride, were supplied by Sinopharm Chemical Reagent Co., Ltd., Shanghai, China, and were used without further treatment.

### 2.2. Membrane Preparation

To prepare the hyperbranched/S-SEBS membranes, S-SEBS was dissolved in 85/15 *w*/*w* THF and hexanol to obtain a viscous solution with 8 wt% of S-SEBS, and different weight percentages of hyperbranched polymers with respect to S-SEBS were added to the solution, i.e., H302:S-SEBS=0.05:1, 0.10:1, 0.15:1, 0.20:1, 0.25:1 and 0.30:1 (mass ratio). The membranes were prepared via solvent evaporation method on a polyfluortetrathylene plate in open air, and then treated in a vacuum oven at 50 °C for 12 h to remove the residual solvent. To prepare the thermally crosslinked membranes, the aforementioned membranes were further heat-treated in a vacuum oven under different heating temperatures from 100 to 130 °C for 2 h. The thickness of the prepared membranes was about 35–40 μm.

### 2.3. Membrane Characterization

The chemical composition and structure of prepared membranes were studied by Fourier transform infrared spectra (FTIR) and element analysis. Specially, a FTIR spectrometer (Nicolet iS50, Yingmei Electronic Technology Co., Ltd., Xi’an, China) was used to confirm the presence of hyperbranched polymers (H302) with spectra wavelengths varying from 4000 to 500 cm^−1^; an elemental analyzer (VARIO EL Ⅲ ELEMENT, Elementar, Frankfurt, Germany) was used to determine the content of carbon, hydrogen and sulfur in the membranes.

The surface morphology of the membranes was observed by scanning electron microscopy (SEM, JSM-6390A, JEOL, Tokyo, Japan) and transmission electron microscope (TEM, G2F30, FEI, Oregon, USA) with an accelerating voltage of 300 kV. Small angle X-ray scattering (SAXS) analysis was performed to characterize the microphase separation structure of the membrane using a SAXSess-MC2 X small angle X-ray scattering meter (Anton Paar, Graz, Austria) with the measurable scattering vector (q) varying from 0.05 to 1.0 nm^−1^. 

Membrane hydrophilicity was tested in terms of water contact angle and water uptake. The content of sulfonated groups that affected the hydrophilicity of the membrane was analyzed by ion exchange capacity (IEC). The IEC was measured via the widely used titration method: approximately 0.01 g of dry membrane was immersed in 0.1 mol/L NaCl aqueous solution at room temperature for two days to liberate the H^+^, which was completely replaced by Na^+^. The solution was then titrated with 0.02 mol/L NaOH aqueous solution with phenolphthalein as an indicator. The water contact angle on the membrane surface was measured by the sessile drop method using a contact angle meter (DSA100, Krüss, Germany), and its change with time on the membrane was captured by video mode to observe the dynamic wetting process of the drops of water. The water uptake of the membrane was calculated according to the equation as follows:

(1) $$\mathrm{Water}\;\mathrm{Uptake}\;=\frac{W_{\mathrm{wet}}{-W}_{\mathrm{dry}}}{W_{\mathrm{dry}}}\times 100\%$$

where *W_dry_* is the weight of the dried membrane and *W_wet_* the weight of the wet membrane after immersion in deionized water at room temperature for 24 h. Every sample was measured three times and the average value was recorded. The experimental error for each sample was ±20%.

The swelling ratio (SR) of the membrane was measured to investigate the dimensional stability of the membrane. The SR was tested in the plane direction of the membrane by immersing a rectangular sample (2 cm × 2 cm) into DI water and calculating using Equation (2) [22]:

(2) $$\mathrm{Swelling}\;\mathrm{Ratio}\;=\frac{L_{\mathrm{wet}}{-L}_{\mathrm{dry}}}{L_{\mathrm{dry}}}\times 100\%$$

where *L_wet_*_,_ and *L_dry_* are the length of the wet and dry membrane, respectively.

The mechanical stability of the membranes (50 mm × 10 mm) was investigated by a tensile strength test using an electronic tensile machine (CMT-6503, Changchun Machinery Academy of Sciences Co., Ltd., Changchun, China) at room temperature with a cross-head speed of 20 mm/min. The thermal stability of membrane was tested by thermo-gravimetric analysis (TGA) (Netzsch STA49F5, Netzsch, Freistaat Bayern, Germany) with a heating rate of 20 °C/min in a nitrogen atmosphere from 50 °C to 550 °C and differential scanning caborimetry (DSC) (TA Instruments, New Castle, DE, USA), with the same heating rate from room temperature to 100 °C. In the DSC analysis, all samples underwent a second heating run to diminish the influence of the remnant water and solvent in the membrane.

### 2.4. Pervaporation Test

**Figure 1:**
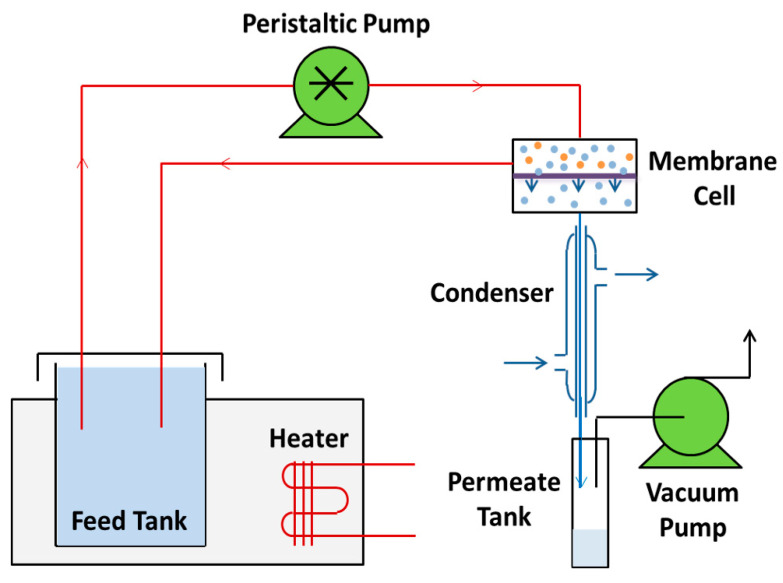
Schematic of pervaporation testing unit.

PV desalination performance was tested on a laboratory-scale device as described in our previous research (Figure 1) [12]. The membrane placed in the metal membrane cell had an effective surface area of 0.001963 m^2^. Feed solution (5 wt% NaCl aqueous solution) was heated to 65 °C and circulated through the membrane cell at a flow rate of 16 L/h using a peristaltic pump. The permeate side was maintained at 6 kPa by a vacuum pump.

The PV desalination performance was evaluated by water flux and salt rejection. The water flux, which refers to the mass of water *M* (kg) permeating through the membrane with a defined area *A* (m^2^) during a certain period time *t* (h), can be calculated based on the equation listed below:

(3) $$J=\frac{M}{A\times t}$$

The salt rejection *R* (%) was obtained by conductivity meter and calculated by the following equation:

(4) $$R=\frac{C_{f}{-C}_{p}}{C_{f}}\times 100\%$$

where *C_f_* and *C_p_* are the salt concentrations in the feed and the permeate solutions, respectively, measured from solution conductivity.

The PV test was repeated at least three times with the error of water flux within ±1% and the standard deviation within 0.06. 

## 3. Results and Discussion

### 3.1. Characterization of H302/S-SEBS Membrane

#### 3.1.1. Crosslinking Reaction

**Figure 2:**
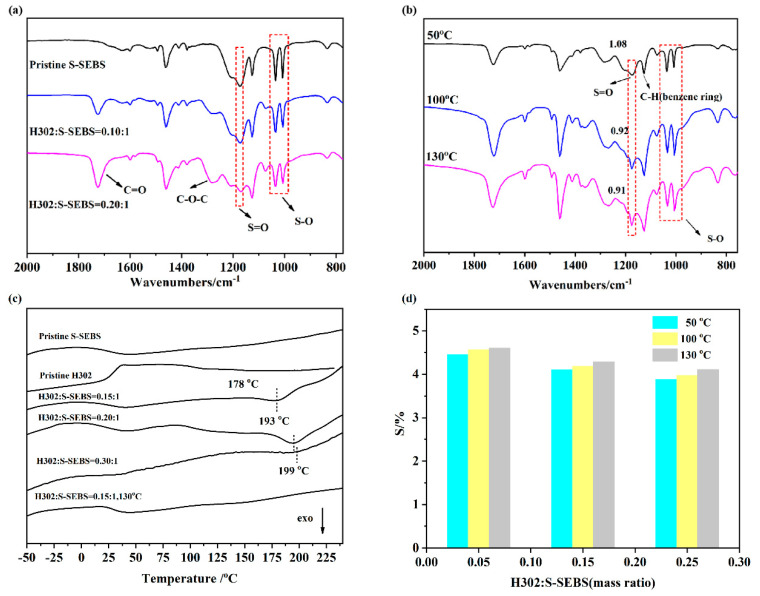
FTIR spectra of H302/S-SEBS membranes (**a**) without thermal treatment; (**b**) with thermal treatment (H302:S-SEBS = 0.15:1); (**c**) DSC of membranes with different contents of H302; (**d**) change of sulfur content in H302/S-SEBS membranes after thermal treatment.

The presence of H302 is evidenced by the FTIR spectra shown in Figure 2a,b. The characteristic peaks of S-SEBS are clearly present in both the pristine and crosslinked membranes, including asymmetric stretching vibrations of SO at 1250–1150 cm^−1^, absorption peaks of SO at 1100–1000 cm^−1^ and stretching vibrations of phenyl rings substituted with sulfonic groups at paraposition, i.e., at 830 cm^−1^. Noticeably, two new peaks appeared in crosslinked membranes after adding hyperbranched polyester H302. The bands located at 1700 cm^−1^ and 1280 cm^−1^ were attributed to the stretching vibration of C=O and C-O-C, respectively, which proved the existence of ester groups. The result indicated that the hyperbranched polyester H302 had been successfully incorporated into the membrane. 

Interestingly, as the thermal treatment temperature increased, the relative intensity of the S=O and S-O signals weakened compared with that of C-H vibration in benzene, located at approximately 1126 cm^−1^. The weakness of the intensity may be attributed to the consumption of sulfonate groups during thermal crosslinking. The weakened SO_3_ peaks indicated the existence of crosslinking between SO_3_ groups of S-SEBS and hydroxyls of H302 or desulfonation. However, according to TG analysis and former reports, desulfonation was not possible at temperatures under 150 °C [12,23]. Thus, the weakness of the SO_3_ peaks suggested interaction between H302 and S-SEBS. 

The crosslinking reaction between H302 and S-SEBS was further confirmed by DSC and the results are listed in Figure 2c. An exothermic peak located in the range of 178–199 °C can be observed for all the S-SEBS/H302 membranes, which was not detected in pristine S-SEBS or H302. Thus, it can be deduced that the exothermic process was related to the crosslinking reaction between H302 and S-SEBS. In addition, the peak position moved to a higher temperature as H302 increased, which probably originated from the H-bond between H302 molecules, hindering the crosslinking reaction between H302 and S-SEBS, and thus leading to a greater heating temperature needed for crosslinking. Moreover, when the S-SEBS/H302 membranes were further treated at 130 °C, the exothermic peak located above 170 °C disappeared, indicating that the reaction between -SO_3_H and –OH was complete after being heated at 130 °C. 

**Figure 3:**
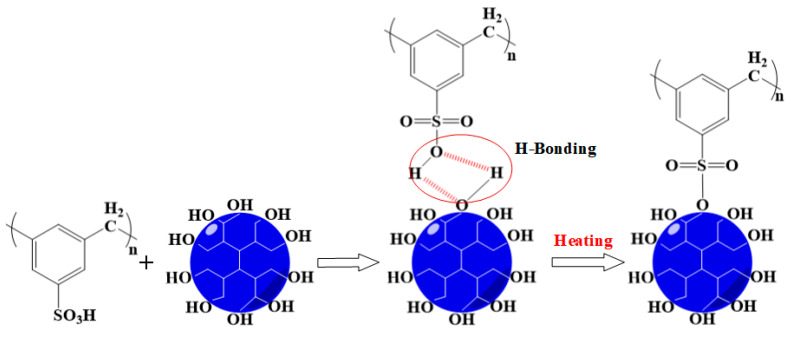
Mechanism of thermal crosslinking.

Elemental analysis was used to trace the change of elemental sulfur content (Cs) during the crosslinking process; the results are given in Figure 2d. With the increase of H302 content in the membrane, the relative content of sulfur decreased. However, as the temperature went up, Cs increased moderately. Since the added H302 did not contain sulfur, and this element is stable in S-SEBS at the crosslinking temperature, the slight increase of sulfur content was attributed to the reduction of membrane mass due to the loss of water molecules during the crosslinking between H302 and S-SEBS, via a mechanism like that shown in Figure 3.

#### 3.1.2. Microstructure of Membranes

**Figure 4:**
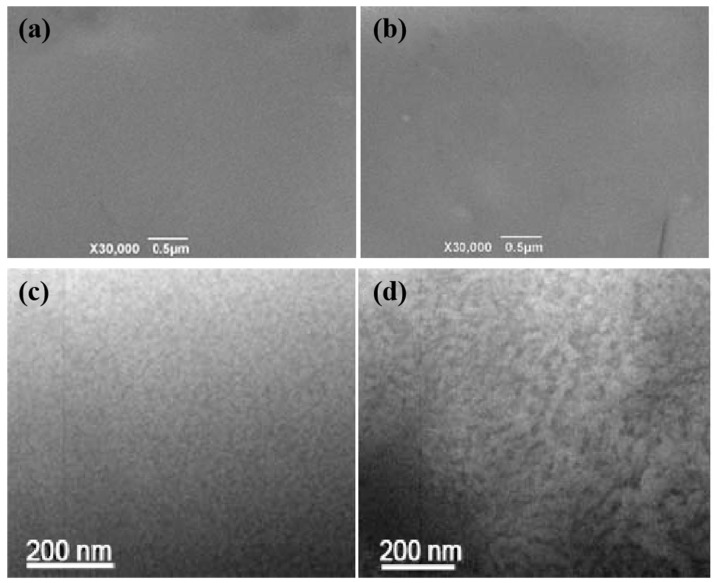
SEM (**a**,**b**) and TEM (**c**,**d**) images of (**a**,**c**) pristine S-SEBS and (**b**,**d**) H302:S-SEBS = 0.30:1 membrane.

The SEM images of the membrane surface shown in Figure 4a,b reveal that both pristine and crosslinked membranes have homogenous and dense structures. The TEM images in Figure 4c,d display dark regions (~10 nm) and bright areas (~30 nm) that may be assigned to the hydrophilic and hydrophobic domains of the membrane, respectively [24,25]. This phenomenon was consistent with the observation by Jung et al. [26]. Notably, the membranes added with H302 exhibited more distinct microphase separation structures compared to the pristine membrane.

**Figure 5:**
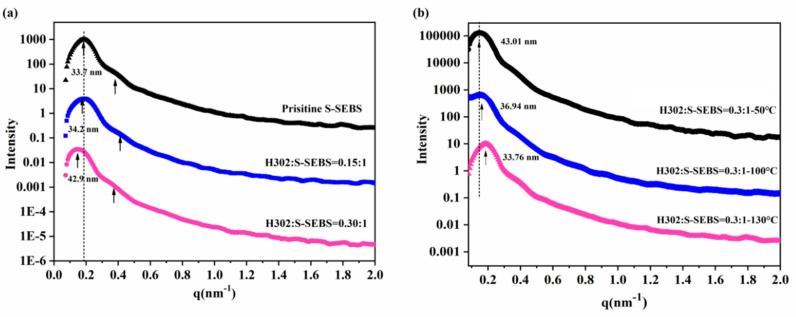
SAXS profiles of H302/S-SEBS membranes (**a**) without thermal treatment (**b**) with thermal treatment.

SAXS was employed to characterize the microphase separation of polymers by detecting the electron density. The SAXS profiles of S-SEBS membranes with different contents of H302 and crosslinked under different temperatures are presented in Figure 5. In Figure 5a, for the pristine S-SEBS membrane, there were two obvious peaks located at q = 0.186 and 0.406 nm^−1^ in the SAXS profile, indicating separate microphases at the nanoscale. According to Bragg’s law (*d* = 2π/q_1_), where q_1_ represents the scattering vector of the first-order peak, the interdomain spacings (*d*) were calculated to be 33.7 nm and 15.5 nm, respectively. It was observed that the incorporation of H302 induced the shift of the first-order peak towards a lower scattering vector. When increasing the content of H302, the shift was more intensive, which reflected the enlargement of hydrophilic domains by the hyperbranched polyester H302. It is known that there is a positive correlation between the interdomain spacing d and the thickness of the hydrophilic domain L, as listed below [27]:

(5) $$L\propto {d\;(1-\varphi }_{\mathrm{EB}})$$

where *L* is the thickness of the hydrophilic domain and *φ_EB_* is the volume ratio of hydrophobic segment of block polymer. As *φ_EB_* remains constant and the hydrophobic domain maintains stability, *L* increases with *d*, which means that the larger the *d*, the thicker the *L*. 

Meanwhile, although this structural change made the channel wider and was beneficial for the transfer of water molecules, it might also cause larger swelling, so further crosslinking with heat treatment was necessary for membrane structural reinforcement. In Figure 5b, as the heat treatment temperature increased, q_1_ of the S-SEBS/H302 membranes moved to a higher vector, indicating a smaller hydrophilic domain. The shrinkage of the hydrophilic domain originated from the dense crosslinking network generated by the crosslinking reaction enhanced with thermal treatment. This change makes the inner structure of the membrane denser, the mechanical strength greater, and at the same time, reduces the size of the channels in the membrane.

#### 3.1.3. Hydrophilicity: Water Contact Angle, IEC, Water Uptake and Swelling Ratio

**Figure 6:**
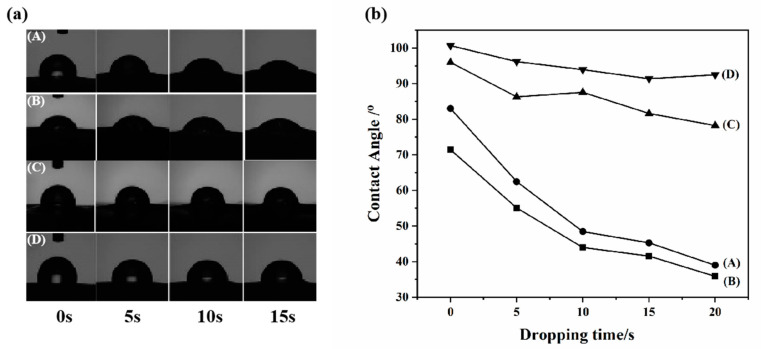
Water contact angle measurement for membranes. (**a**) Water droplets on membrane surface and (**b**) water contact angle with drop age (A: pristine S-SEBS; B: H302:S-SEBS = 0.15:1; C: H302:S-SEBS = 0.15:1 crosslinked at 100 °C; D: H302:S-SEBS = 0.15:1 crosslinked at 130 °C).

The water contact angle was tested to characterize the surface hydrophilic properties of the membrane. The curves of the water contact angle as a function of time for H302/S-SEBS membranes with and without thermal treatment are shown in Figure 6. For the pristine S-SEBS membrane, the initial contact angle was approximately 75° and dropped rapidly to 35° within 20 s. The contact angle hysteresis phenomenon (Figure 6a) was associated with the interfacial composition of the polymer [28,29]. When the S-SEBS membrane surface was exposed to water, the hydrophilic sulfonic groups were gradually drawn to the surface as water diffused into the membrane, thus lowering the contact angle. Moreover, membrane surface buckling occurred distinctively because of membrane local swelling after water absorption. Compared to the pristine membrane, the H302/S-SEBS membrane demonstrated a lower contact angle, as well as a faster permeation of water droplets on the surface (Figure 6b). Since the hyperbranched molecules H302 provided the hydrophilic functional groups to the membrane, the H302/S-SEBS membrane exhibited increased hydrophilic properties. Thermal treatment significantly increased the contact angles of H302/S-SEBS membranes and a higher heating temperature led to a higher contact angle (Figure 6b–d). In addition, the contact angle became more stable with time. This was associated with the consumption of–SO_3_H groups during crosslinking, which significantly lowered the hydrophilicity of the membranes.

**Figure 7:**
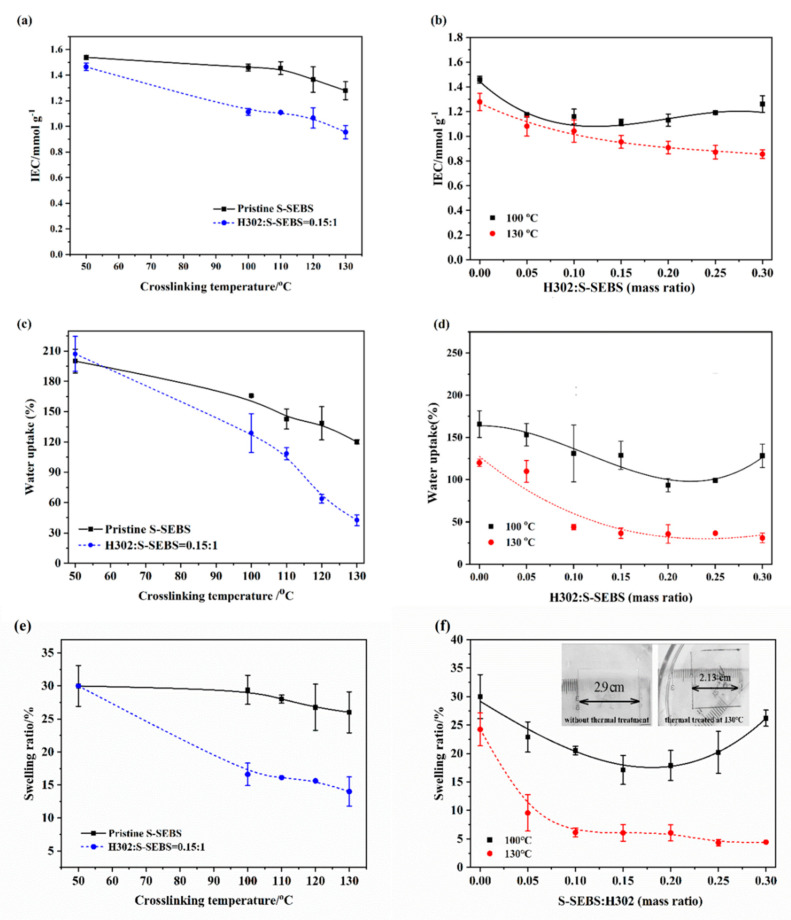
IEC (**a**,**b**), water uptake (**c**,**d**) and swelling ratio (**e**,**f**) of membranes as a function of crosslinking temperature and H302 content.

**Figure 8:**
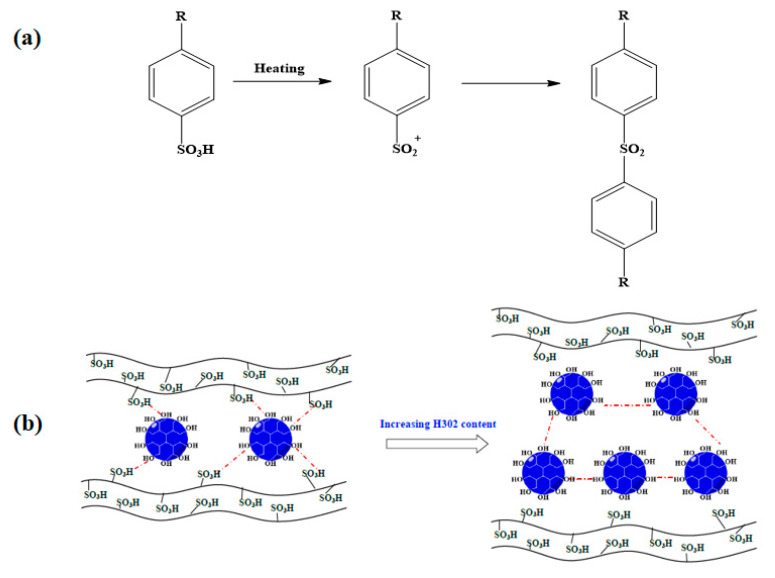
Schematic diagram of (**a**) thermal-crosslinking of S-SEBS without crosslinker and (**b**) H-bonding interaction between H302 in thermal-crosslinking with S-SEBS.

The hydrophilicity of the membrane can also be well reflected by the water uptake of the membrane, which plays an important role in water flux across the membrane. The water uptake of the membrane was affected by both the content of functional groups and the microstructure of the membrane for water absorption. The content of sulfonic acid groups can be readily identified by the IEC level. The influence of H302 content and crosslinking temperature on IEC are shown in Figure 7a,b. Figure 7a indicates that for pristine S-SEBS membranes, IEC decreased with heat treatment and showed greater decline at higher temperatures. This was because that pristine sulfonated membranes could form sulfonate esters by the condensation of sulfonic acid functionalities [30], as illustrated in Figure 8a. By comparison, it was obvious that the addition of H302 reduced the IEC value more significantly compared to single thermal treatment. This can be explained by the fact that there was less chain entanglement in hyperbranched polymers and the abundant terminal functional groups endowed H302 with higher reactivity to initiate the crosslinking reaction. Figure 7b illustrates that under a mild crosslinking condition (i.e., 100 °C), the IEC value first decreased and then increased slightly with the increase of H302 content from approximately 0.15:1. As the hyperbranched polyester used here was terminated by hydroxyl groups, either interspecies interactions between H302 and S-SEBS or intramolecular interactions between H302 molecules may occur. Thus, it can be deduced that below 0.15:1, interspecies interactions between hydroxyls of H302 and sulfonate groups of S-SEBS prevailed, consuming more sulfonated groups. However, as illustrated in Figure 8b, when the H302 content further increased, intramolecular interactions between H302 molecules dominated, hindering the interaction between S-SEBS and H302. For H302/S-SEBS membranes crosslinked at 130 °C (Figure 7b), the IEC value continuously decreased with H302 content; this may have been because a higher crosslinking temperature could break the H-bond interaction between H302 molecules. These results indicated that increasing the heating temperature and H302 content are both beneficial to the crosslinking reaction. 

It can be observed that the water uptake of hyperbranched-S-SEBS membranes in Figure 7c,d presented a similar trend with IEC value as function of H302 contents and heating temperature. In Figure 7c, the water uptake of the pristine S-SEBS and H302/S-SEBS membranes was significantly reduced after thermal treating, and that of the H302/S-SEBS membranes decreased faster than the pristine film. The membranes with 0.15:1 of H302 showed a dramatic decrease of water uptake from 200% to a mere 20% as the treating temperature increased from 100 °C to 130 °C. It was obvious that increasing the temperature could intensify the interaction between H302 and S-SEBS, thus lowering the hydrophilicity of the membranes. In addition, as observed in the SAXS diagram (Figure 5b), the interdomain d-spacings reduced significantly with the increase of heating temperature, indicating that the hydrophilic domain shrank and the water transport was hindered. Both the decreases of functional group content and the size of hydrophilic domain could induce a dramatic reduction of water uptake. It is known that the water uptake of the membrane is closely related to water diffusion in the membrane; therefore, the water flux of the membrane would have a considerable decline. To obtain high flux, it is important to choose an appropriate heating temperature.

Figure 7d illustrates that for membranes treated at 100 °C, the water-uptake first dropped and then rose with the increase of H302 amount, which was similar to the IEC trend in Figure 7b. It is apparent that increasing the amount of H302 allowed more hydroxyls to participate in the crosslinking reactions with sulfonated groups of S-SEBS, which consumed more hydrophilic groups, leading to the reduction of water uptake. However, as the H302 amount further increased, intramolecular interactions between H302 dominated, hindering the interaction between S-SEBS and H302. Thus, reducing the crosslink-degree of H302/S-SEBS film permits more water to be absorbed in the membrane, leading to the return of water uptake. Meanwhile, for the membranes treated above 130 °C, the water uptake first decreased and then remained unchanged, as a higher crosslinking temperature could break the H-bond interactions between H302 molecules; therefore, the water uptake would not be promoted, even if the H302 content gradually increased.

Figure 7e,f demonstrates the swelling ratio of S-SEBS membranes in water. Figure 7e indicates that H302/S-SEBS membranes have a much lower swelling ratio, and that the anti-swelling capability increases with the heating temperature, thereby facilitating the building of a denser network and improving the dimensional stability of the membrane. Figure 7f indicates that under a mild crosslinking condition (i.e., 100 °C), the swelling ratio first decreased and then increased slightly with an increase of H302 content, minimizing at 0.15:1. However, when the membranes were thermally treated at 130 °C, the swelling ratio monotonously decreased with H302 content. The reason for this was that below 100 °C, with an increase of H302 content, the hydrophilic regions expanded and more water molecules could be accommodated, making the swelling degree level rebound. Meanwhile, at 130 °C, the higher crosslinking temperature helped to break the hydrogen bond between H302, making the network denser and further limiting the mobility of the chains [31]. As a result, the swelling ratio value decreased with H302. 

#### 3.1.4. Mechanical and Thermal Properties

**Figure 9:**
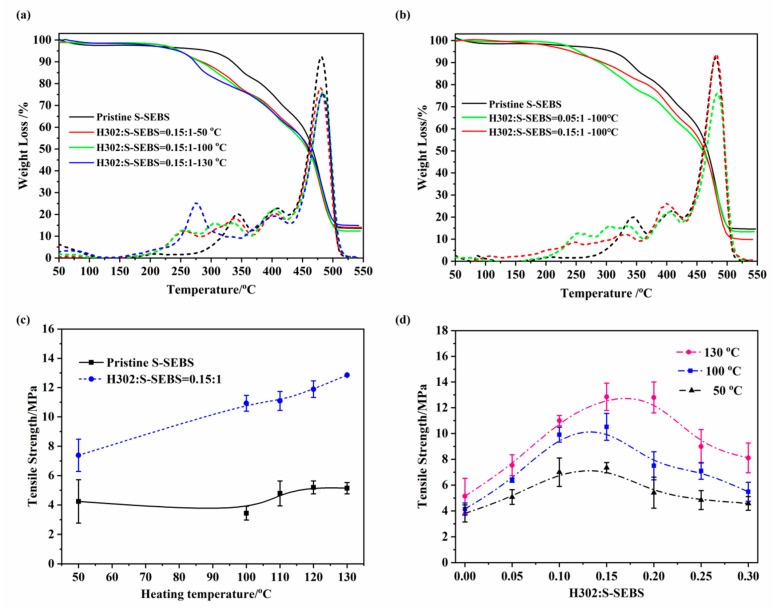
TG (**a**,**b**) and tensile strength (**c**,**d**) of membranes with different treating temperatures and H302 contents.

The TG and DTG curves of H302/S-SEBS membranes crosslinked at different temperatures are shown in Figure 9a,b. Pristine S-SEBS exhibited two weight loss stages in the test range. The first stage was located between 300 °C and 400 °C, which was attributed to the desulfonation process. The second stage was observed above 480 °C, associated with the polymer backbone degradation. Apart from the two stages, an extra one appeared at approximately 250 °C for H302/S-SEBS membranes, which originated from the degradation of H302. By increasing the crosslinking temperature, the H302 degradation temperature increased from 250 °C to 275 °C, indicating an enhanced thermal stability after thermal crosslinking (Figure 9a). Furthermore, with the increasing of H302 content, this degradation temperature increased from 250 °C to 260 °C, indicating an enhanced thermal stability after adding more crosslinkers (Figure 9b). 

The tensile strength results of the H302/S-SEBS membranes are shown in Figure 9c,d. Figure 9c illustrates that compared with the pristine S-SEBS membrane, the crosslinked membranes demonstrated much higher tensile strength, indicating improved mechanical stability. Increasing the treating temperature from 50 °C to 130 °C led to a continuous sharp increase in tensile strength, up to 67%. The tensile strength could be increased by 140–200% compared to the pristine membrane by heating treatment at 100 °C to 130 °C. As mentioned in the previous section, the hydrogen bonding between the hydroxyl groups of H302 and the sulfonate groups of S-SEBS may have formed a network structure, contributing to higher tensile strength. However, excessive H302 molecules tended to form intramolecular interactions, leading to a gradual weakening of tensile strength. As can be seen in Figure 9d, when the H302 ratio was below 0.15:1, the tensile strength went up continuously for the interspecies hydrogen between hydroxyls of H302, and sulfonate groups of S-SEBS were the prevailing interaction, forming a stronger network structure within the membrane. However, as the H302 content further increased, intramolecular hydrogen bonds between H302 molecules dominated, and the tensile strength showed a downward trend.

### 3.2. Pervaporation Performance

**Figure 10:**
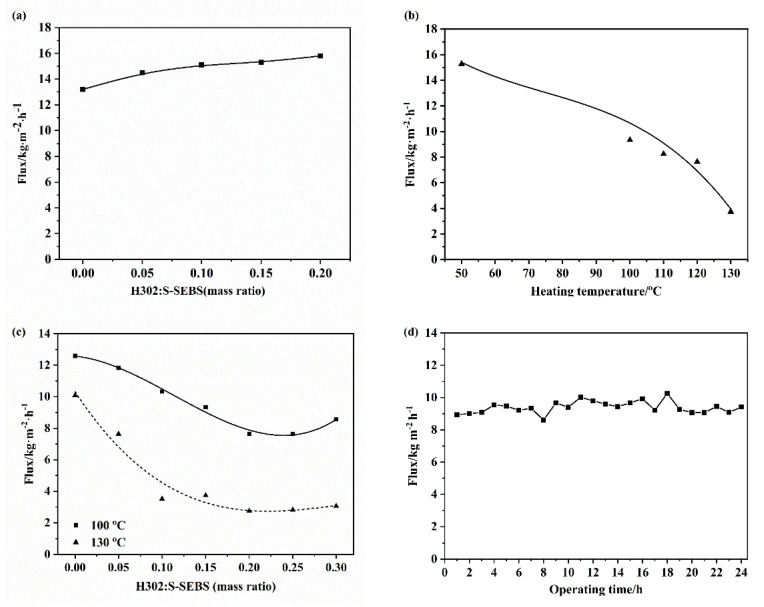
Pervaporation performance of (**a**) without thermal treatment; (**b**) with thermal treatment at different temperatures (H302: S-SEBS = 0.15:1); (**c**) at different mass ratios of H302: S-SEBS; (**d**) stability test for membrane (H302:S-SEBS = 0.15:1, thermal treatment at 100 °C). (All PV test with 5 wt% NaCl feed solution at 65 °C).

Figure 10a shows the pervaporation performance of H302/S-SEBS membranes with different amounts of H302 when treating 5 wt% hypersaline water. A salt rejection of 99.9% was achieved for all the membranes. The water flux generally increased with the H302 content due to increased hydrophilicity, as shown by the contact angle and broadened hydrophilic domain in the SAXS results. However, a further increase in H302 content may result in excessive swelling and affect the long-term stability, which was supported by the decreased tensile strength above a mass ratio (H302:S-SEBS) of 0.20:1. 

Figure 10b shows the water flux and salt rejection of H302/S-SEBS membranes heat treated at different temperatures. The flux was closely associated with the content of hydrophilic groups and the size of the hydrophilic domain. It is obvious that the larger the IEC, the higher the flux. For pristine S-SEBS, thermal crosslinking hardly changed the IEC value; thus, the flux only exhibited a moderate decline. For the H302/S-SEBS membrane (H302:S-SEBS = 0.15:1), increasing the heat treatment temperature enhanced the crosslinking reaction, resulting in a denser microstructure, lower IEC and lower hydrophilicity. As a result, the flux demonstrated a continuous reduction from 15.2 kg·m^−2^·h^−1^ to 3.8 kg·m^−2^·h^−1^ when the heat treatment temperature increased from 50 °C to 130 °C. It is worth noting that the membrane heat treated at 100 °C maintained a flux of 9.3 kg·m^−2^·h^−1^.

Figure 10c illustrates that the water flux of the membrane first dropped and then had a slight rise with the increase of H302 amount, which was different from the trend depicted in Figure 10a. This was because the effect of thermal treatment is associated with H302 amount. As mentioned above, increasing the amount of H302 allowed more hydroxyls to participate in the crosslinking reactions with sulfonate groups of S-SEBS, which consumed more hydrophilic groups, leading to a reduction of IEC and water-uptake, and thus, also of the membrane flux. However, as the H302 amount further increased, the larger hydrophilic domain and free volume of the mixed membrane compensated for the loss of hydrophilic groups, permitting more water to be absorbed in the membrane, which led to the recovery of the flux. 

Figure 10d indicates that the H302/S-SEBS membrane (H302:S-SEBS = 0.15:1, thermally treated at 100 °C) showed stable performance in an elongated running. The salt rejection of the membrane always remained above 99.8%, and there was no obvious decline in water flux, proving that the hyperbranched-S-SEBS membranes had great stability. 

Furthermore, the hyperbranched-S-SEBS membrane exhibited a high degree of salt rejection, irrespective of heating temperature and H302 content, as shown in Figure 10a–d. This was mainly because of the intrinsic density feature and charge exclusion effect of the PV membrane, as well as the nonvolatile nature of salt.

## 4. Conclusions

To improve the mechanical properties of sulfonated styrene-ethylene/butylene-styrene (S-SEBS) polymer membranes with a high sulfonation degree, and to maintain high water flux, commercially available hydroxyl-terminated hyperbranched polyester (H302) was introduced to crosslink a S-SEBS polymer membrane. When the hydroxyl-terminated hyperbranched polyester (H302) was simply blended with S-SEBS polymer through hydrogen bonding interactions, small-angle X-ray scattering (SAXS) indicated that the introduction of hyperbranched polyester enlarged the hydrophilic microdomain. As a result, the water flux significantly increased compared with that of the pristine membrane. Meanwhile, the formation of a hydrogen bonding network improved the membrane tensile strength by 87%. However, the swelling degree of the membrane increased with the increase of water-uptake. To enhance the stability of the crosslinking network, thermal treatment was further employed. Thermal crosslinking was confirmed by Fourier transform infrared spectroscopy (FTIR), differential scanning calorimetry (DSC) and elemental analysis (EA). The denser network increased the tensile strength by 140–200% and reduced the swelling ratio by 45–70%, while still maintaining reasonable water flux. When treating 5 wt% hypersaline water, the crosslinked membrane with 15 wt% H302 treated at 100 °C reached a flux of 9.34 kg·m^−2^·h^−1^ and a salt rejection of 99.9%. The results indicate that the hyperbranched-S-SEBS membrane is promising for use in PV desalination.
